# 2-(4-Meth­oxy­phen­yl)-1-phenyl-1*H*-benzimidazole

**DOI:** 10.1107/S1600536813001566

**Published:** 2013-01-23

**Authors:** T. Mohandas, K. Jayamoorthy, J. Jayabharathi, P. Sakthivel

**Affiliations:** a Shri Angalamman College of Engineering and Technology, Siruganoor, Tiruchirappalli 621 105, India; b Department of Chemistry, Annamalai University, Annamalainagar 608 002, Tamilnadu, India; c Department of Physics, Urumu Dhanalakshmi College, Tiruchirappalli 620 019, India

## Abstract

In the title compound, C_20_H_16_N_2_O, the 1*H*-benzimidazole ring forms dihedral angles of 48.00 (6) and 64.48 (6)°, respectively with the benzene and phenyl rings, which are inclined to one another by 58.51 (7)°. In the crystal, weak C—H⋯π inter­actions are the only inter­molecular inter­actions present.

## Related literature

For background to benzimidazole derivatives, see: Mason *et al.* (1999 ▶). For their biological activities such as anti­microbial & anti­cancer, anti­diabetic, anti­fungal, anti HIV and anti­viral, see: Demirayak *et al.* (2002 ▶); Minoura *et al.* (2004 ▶); Pawar *et al.* (2004 ▶); Rao *et al.* (2003 ▶); Tomei *et al.* (2003 ▶). For their action as polymerase and transcriptase inhibitors, see: Beaulieu *et al.* (2004 ▶; Morningstar *et al.* (2007 ▶); Roth *et al.* (1997 ▶); For other related studies, see: Jayabharathi *et al.* (2012 ▶)
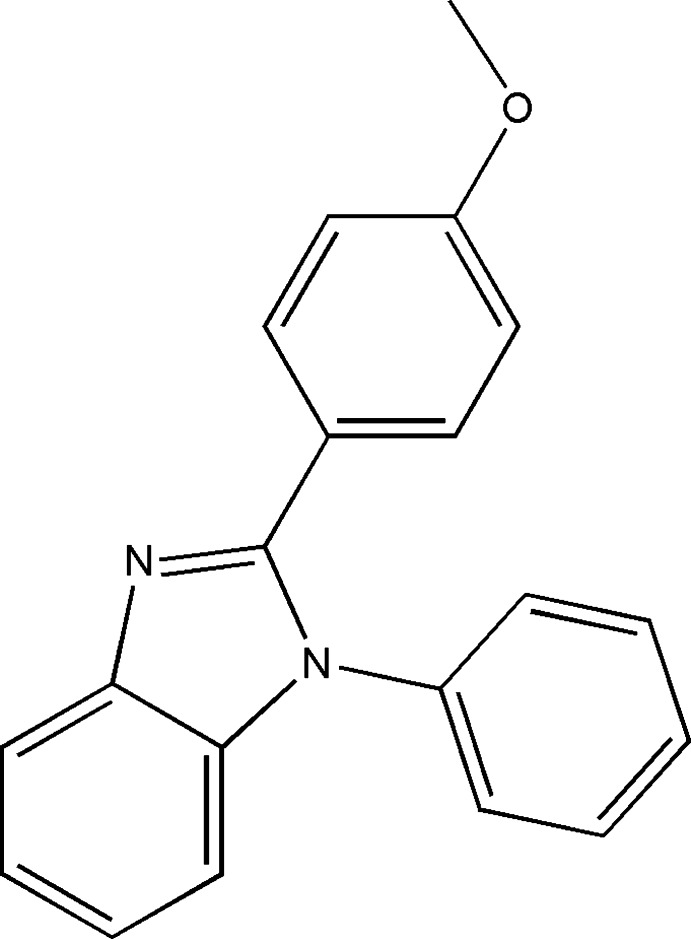


## Experimental

### 

#### Crystal data

C_20_H_16_N_2_O
*M* *_r_* = 300.35
Monoclinic, 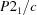

*a* = 12.3220 (3) Å
*b* = 7.3030 (2) Å
*c* = 18.2450 (3) Å
β = 108.909 (1)°
*V* = 1553.22 (6) Å^3^
*Z* = 4
Mo *K*α radiation
μ = 0.08 mm^−1^
*T* = 293 K
0.30 × 0.30 × 0.20 mm

#### Data collection

Bruker Kappa APEXII CCD diffractometer
Absorption correction: multi-scan (*SADABS*; Bruker, 2008 ▶) *T*_min_ = 0.956, *T*_max_ = 0.999
13787 measured reflections
2728 independent reflections
2283 reflections with *I* > 2σ(*I*)
*R*_int_ = 0.024

#### Refinement

*R*[*F* ^2^ > 2σ(*F* ^2^)] = 0.035
*wR*(*F* ^2^) = 0.092
*S* = 1.03
2728 reflections
209 parameters
H-atom parameters constrained
Δρ_max_ = 0.11 e Å^−3^
Δρ_min_ = −0.21 e Å^−3^

### 

Data collection: *APEX2* (Bruker, 2008 ▶); cell refinement: *APEX2* and *SAINT* (Bruker, 2008 ▶); data reduction: *SAINT*; program(s) used to solve structure: *SHELXS97* (Sheldrick, 2008 ▶); program(s) used to refine structure: *SHELXL97* (Sheldrick, 2008 ▶); molecular graphics: *ORTEP-3* (Farrugia, 2012 ▶); software used to prepare material for publication: *PLATON* (Spek, 2009 ▶).

## Supplementary Material

Crystal structure: contains datablock(s) global, I. DOI: 10.1107/S1600536813001566/go2077sup1.cif

Structure factors: contains datablock(s) I. DOI: 10.1107/S1600536813001566/go2077Isup2.hkl

Supplementary material file. DOI: 10.1107/S1600536813001566/go2077Isup3.cml

Additional supplementary materials:  crystallographic information; 3D view; checkCIF report

## Figures and Tables

**Table 1:** Hydrogen-bond geometry (Å, °) *Cg*2 and *Cg*3 are the centroids of the C2–C7 and C9–C14 phenyl rings, respectively.

*D*—H⋯*A*	*D*—H	H⋯*A*	*D*⋯*A*	*D*—H⋯*A*
C6—H6⋯*Cg*2^i^	0.93	2.86	3.5361 (15)	130
C13—H13⋯*Cg*3^ii^	0.93	2.83	3.4594 (16)	126

Symmetry codes: (i) 
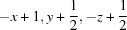
; (ii) 
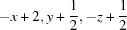
.

**Fig. 1.**
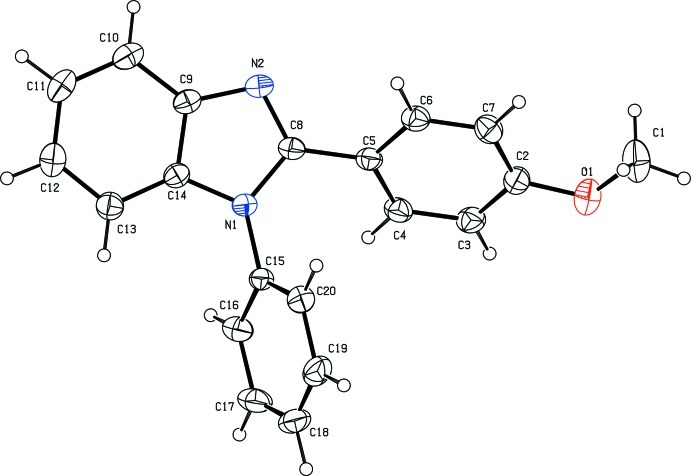
The molecular structure and labelling scheme for (I) with displacement ellipsoids for non-H atoms are drawn at the 30% probability level.

**Fig. 2.**
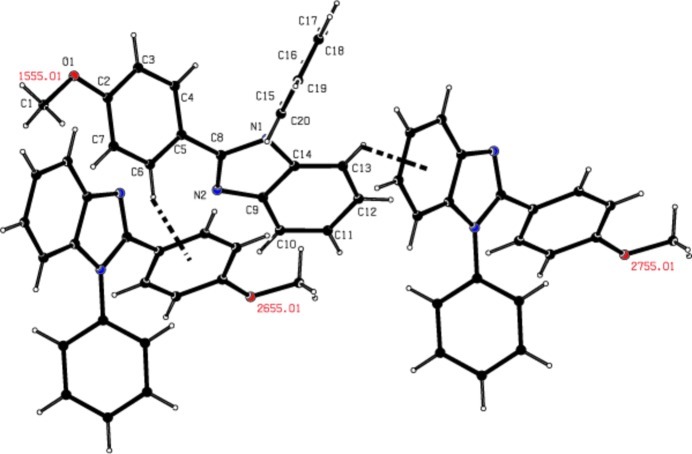
A packing diagram for (I) is shown. Intermolecular hydrogen bonds are shown as dashed lines.

## supplementary crystallographic information

### Comment

The benzimidazole core is classified by medicinal chemists as one of the 'privileged substructures' for drug design,in light of the affinity they display towards a variety of enzymes and protein receptors (Mason *et al.*,1999).

The synthesis of benzimidazoles has received much attention owing to the varied biological activity such as antifungal (Pawar *et al.*, 2004), antiviral (Tomei *et al.*, 2003), antiHIV (Rao *et al.*, 2003), antidiabetic (Minoura *et al.*, 2004), antimicrobial and anticancer (Demirayak *et al.*, 2002), properties exhibited by a number of derivatives of these compounds.

Benzimidazole derivatives possess antioxidant activities (Jayabharathi *et al.*,2012).

They have emerged as potent non nucleoside inhibitors of HIV-1 reverse transcriptase (Roth *et al.*,1997, Morningstar *et al.*,2007)

It also acts as a specific inhibitors of the NS5B polymerase of the hepatitis C virus (HCV) (Beaulieu, *et al.*, 2004).

The molecular structure of (I), is shown in Fig.1. The (N1/N2/C8—C14) 1*H*-benzimidazole ring is planar. It forms dihedral angles of 48.00 (6)° and 64.48 (6)° with the mean planes of the C2—C7 and C15—C20 phenyl rings respectively.

The C1—O1—C2—C7 and C3—C2—O1—C1 torsion angles are 2.8 (2)° and -176.42 (14)° respectively.

A C6—H6···π interaction involving the phenyl ring of methoxybenzene at the symmetry code [1 - *x*,1/2 + *y*,1/2 - *z*] and C13—H13···π interaction involving the benzene ring of benzimidazole at the symmetry code [2 - *x*,1/2 + *y*,1/2 - *z*] are also found.

### Experimental

To pure *N*-phenyl-0-phenylenediamine(17 mmol,3.128 g) in ethanol(10 ml), 4-methoxy benzaldehyde(17 mmol,2.1 ml) and ammonium acetate(3 g) was added for about 1 h while maintaining the temperature at 80°C. The reaction mixture was refluxed and the completion of reaction was monitored by TLC, finally the reactants extracted with dichloromethane. The solid separated was purified by column chromatography using petroleum ether as the eluent.Yield:2.65 g(50%) from which it was crystallized.

### Refinement

All the hydrogen atoms were geometrically fixed and allowed to ride on their parent atoms with C—H = 0.93 - 0.97 Å, and *U*_iso_(H) = 1.2Ueq(C).

### Crystal data

**Table:**

C_20_H_16_N_2_O	*F*(000) = 632
*M**_r_* = 300.35	*D*_x_ = 1.284 Mg m^−^^3^
Monoclinic, *P*2_1_/*c*	Mo *K*α radiation, λ = 0.71073 Å
Hall symbol: -P 2ybc	Cell parameters from 6267 reflections
*a* = 12.3220 (3) Å	θ = 2.8–31.8°
*b* = 7.3030 (2) Å	µ = 0.08 mm^−^^1^
*c* = 18.2450 (3) Å	*T* = 293 K
β = 108.909 (1)°	Block, colourless
*V* = 1553.22 (6) Å^3^	0.30 × 0.30 × 0.20 mm
*Z* = 4	

### Data collection

**Table:**

Bruker Kappa APEXII CCD diffractometer	2728 independent reflections
Radiation source: fine-focus sealed tube	2283 reflections with *I* > 2σ(*I*)
Graphite monochromator	*R*_int_ = 0.024
ω and φ scan	θ_max_ = 25.0°, θ_min_ = 2.4°
Absorption correction: multi-scan (*SADABS*; Bruker, 2008)	*h* = −14→14
*T*_min_ = 0.956, *T*_max_ = 0.999	*k* = −8→8
13787 measured reflections	*l* = −21→21

### Refinement

**Table:**

Refinement on *F*^2^	Primary atom site location: structure-invariant direct methods
Least-squares matrix: full	Secondary atom site location: difference Fourier map
*R*[*F*^2^ > 2σ(*F*^2^)] = 0.035	Hydrogen site location: inferred from neighbouring sites
*wR*(*F*^2^) = 0.092	H-atom parameters constrained
*S* = 1.03	*w* = 1/[σ^2^(*F*_o_^2^) + (0.0421*P*)^2^ + 0.3566*P*] where *P* = (*F*_o_^2^ + 2*F*_c_^2^)/3
2728 reflections	(Δ/σ)_max_ = 0.001
209 parameters	Δρ_max_ = 0.11 e Å^−^^3^
0 restraints	Δρ_min_ = −0.21 e Å^−^^3^

### Special details

**Table:**

Geometry. Bond distances, angles *etc*. have been calculated using the rounded fractional coordinates. All e.s.d.'s are estimated from the variances of the (full) variance-covariance matrix. The cell e.s.d.'s are taken into account in the estimation of distances, angles and torsion angles
Refinement. Refinement of *F*^2^ against ALL reflections. The weighted *R*-factor *wR* and goodness of fit *S* are based on *F*^2^, conventional *R*-factors *R* are based on *F*, with *F* set to zero for negative *F*^2^. The threshold expression of *F*^2^ > σ(*F*^2^) is used only for calculating *R*-factors(gt) *etc*. and is not relevant to the choice of reflections for refinement. *R*-factors based on *F*^2^ are statistically about twice as large as those based on *F*, and *R*- factors based on ALL data will be even larger.

### Fractional atomic coordinates and isotropic or equivalent isotropic displacement parameters (Å^2^)

**Table:**

	*x*	*y*	*z*	*U*_iso_*/*U*_eq_	
C1	0.15098 (14)	0.1529 (3)	0.11631 (12)	0.0752 (5)	
H1A	0.1582	0.0917	0.1642	0.113*	
H1B	0.0756	0.1323	0.0803	0.113*	
H1C	0.1629	0.2819	0.1256	0.113*	
C2	0.34701 (11)	0.10389 (19)	0.12724 (8)	0.0448 (3)	
C3	0.42477 (12)	0.0429 (2)	0.09213 (8)	0.0486 (4)	
H3	0.3983	−0.0131	0.0438	0.058*	
C4	0.54039 (12)	0.0648 (2)	0.12827 (7)	0.0450 (3)	
H4	0.5916	0.0255	0.1037	0.054*	
C5	0.58222 (11)	0.14511 (18)	0.20131 (7)	0.0385 (3)	
C6	0.50382 (12)	0.20005 (19)	0.23664 (8)	0.0443 (3)	
H6	0.5302	0.2506	0.2860	0.053*	
C7	0.38733 (12)	0.1813 (2)	0.20007 (8)	0.0477 (3)	
H7	0.3360	0.2209	0.2245	0.057*	
C8	0.70531 (11)	0.17362 (18)	0.24035 (7)	0.0375 (3)	
C9	0.87357 (11)	0.18293 (18)	0.32458 (7)	0.0404 (3)	
C10	0.96985 (13)	0.1720 (2)	0.39081 (8)	0.0513 (4)	
H10	0.9646	0.1228	0.4366	0.062*	
C11	1.07235 (13)	0.2354 (2)	0.38684 (9)	0.0557 (4)	
H11	1.1373	0.2294	0.4306	0.067*	
C12	1.08135 (13)	0.3089 (2)	0.31854 (9)	0.0546 (4)	
H12	1.1523	0.3504	0.3178	0.066*	
C13	0.98813 (12)	0.3218 (2)	0.25235 (8)	0.0468 (3)	
H13	0.9940	0.3716	0.2069	0.056*	
C14	0.88502 (11)	0.25677 (18)	0.25688 (7)	0.0377 (3)	
C15	0.74297 (10)	0.32860 (18)	0.12689 (7)	0.0365 (3)	
C16	0.78572 (13)	0.2560 (2)	0.07224 (7)	0.0478 (4)	
H16	0.8363	0.1576	0.0845	0.057*	
C17	0.75247 (15)	0.3317 (2)	−0.00127 (8)	0.0589 (4)	
H17	0.7807	0.2833	−0.0387	0.071*	
C18	0.67873 (15)	0.4765 (3)	−0.01939 (8)	0.0626 (5)	
H18	0.6565	0.5259	−0.0690	0.075*	
C19	0.63744 (13)	0.5492 (2)	0.03560 (9)	0.0601 (4)	
H19	0.5877	0.6488	0.0233	0.072*	
C20	0.66926 (11)	0.4754 (2)	0.10932 (8)	0.0479 (4)	
H20	0.6411	0.5245	0.1466	0.057*	
O1	0.23425 (8)	0.08312 (16)	0.08496 (6)	0.0636 (3)	
N1	0.77587 (9)	0.24986 (15)	0.20301 (5)	0.0375 (3)	
N2	0.76084 (9)	0.13147 (17)	0.31277 (6)	0.0438 (3)	

### Atomic displacement parameters (Å^2^)

**Table:**

	*U*^11^	*U*^22^	*U*^33^	*U*^12^	*U*^13^	*U*^23^
O1	0.0447 (6)	0.0665 (8)	0.0712 (7)	−0.0034 (5)	0.0074 (5)	−0.0040 (6)
N1	0.0387 (6)	0.0421 (6)	0.0310 (5)	−0.0019 (5)	0.0105 (4)	0.0038 (4)
N2	0.0489 (7)	0.0512 (7)	0.0316 (5)	0.0002 (5)	0.0134 (5)	0.0035 (5)
C1	0.0459 (9)	0.0666 (12)	0.1100 (15)	0.0005 (8)	0.0207 (9)	−0.0018 (11)
C2	0.0426 (7)	0.0394 (8)	0.0483 (8)	−0.0036 (6)	0.0091 (6)	0.0057 (6)
C3	0.0548 (8)	0.0520 (9)	0.0370 (7)	−0.0121 (7)	0.0121 (6)	−0.0045 (6)
C4	0.0504 (8)	0.0507 (9)	0.0382 (7)	−0.0071 (7)	0.0203 (6)	−0.0039 (6)
C5	0.0442 (7)	0.0388 (7)	0.0336 (6)	−0.0036 (6)	0.0142 (5)	0.0030 (5)
C6	0.0505 (8)	0.0461 (8)	0.0377 (7)	−0.0018 (6)	0.0164 (6)	−0.0038 (6)
C7	0.0478 (8)	0.0461 (8)	0.0532 (8)	0.0027 (7)	0.0220 (7)	−0.0007 (7)
C8	0.0443 (7)	0.0389 (7)	0.0320 (6)	−0.0010 (6)	0.0160 (5)	0.0002 (5)
C9	0.0468 (8)	0.0394 (8)	0.0333 (6)	0.0038 (6)	0.0105 (6)	−0.0006 (5)
C10	0.0566 (9)	0.0547 (9)	0.0376 (7)	0.0092 (7)	0.0080 (6)	0.0033 (6)
C11	0.0471 (8)	0.0577 (10)	0.0498 (8)	0.0081 (7)	−0.0015 (7)	−0.0036 (7)
C12	0.0417 (8)	0.0514 (9)	0.0656 (10)	−0.0010 (7)	0.0103 (7)	−0.0015 (8)
C13	0.0460 (8)	0.0442 (8)	0.0496 (8)	−0.0006 (6)	0.0147 (6)	0.0032 (6)
C14	0.0405 (7)	0.0351 (7)	0.0360 (6)	0.0029 (6)	0.0103 (5)	0.0005 (5)
C15	0.0380 (7)	0.0403 (7)	0.0301 (6)	−0.0067 (6)	0.0095 (5)	0.0032 (5)
C16	0.0587 (9)	0.0471 (9)	0.0419 (7)	−0.0050 (7)	0.0225 (7)	0.0000 (6)
C17	0.0781 (11)	0.0663 (11)	0.0376 (8)	−0.0233 (9)	0.0260 (7)	−0.0047 (7)
C18	0.0695 (10)	0.0707 (12)	0.0367 (8)	−0.0261 (9)	0.0024 (7)	0.0136 (8)
C19	0.0506 (9)	0.0592 (10)	0.0594 (9)	−0.0021 (8)	0.0024 (7)	0.0211 (8)
C20	0.0455 (8)	0.0510 (9)	0.0461 (7)	0.0003 (7)	0.0136 (6)	0.0050 (7)

### Geometric parameters (Å, º)

**Table:**

O1—C1	1.421 (2)	C15—C20	1.374 (2)
O1—C2	1.3616 (18)	C16—C17	1.3842 (19)
N1—C8	1.3835 (17)	C17—C18	1.363 (3)
N1—C14	1.3859 (17)	C18—C19	1.371 (2)
N1—C15	1.4349 (15)	C19—C20	1.383 (2)
N2—C8	1.3126 (16)	C1—H1A	0.9599
N2—C9	1.3868 (18)	C1—H1B	0.9599
C2—C3	1.387 (2)	C1—H1C	0.9600
C2—C7	1.380 (2)	C3—H3	0.9301
C3—C4	1.371 (2)	C4—H4	0.9299
C4—C5	1.3930 (18)	C6—H6	0.9300
C5—C6	1.383 (2)	C7—H7	0.9301
C5—C8	1.4666 (19)	C10—H10	0.9301
C6—C7	1.380 (2)	C11—H11	0.9299
C9—C10	1.395 (2)	C12—H12	0.9302
C9—C14	1.3962 (18)	C13—H13	0.9301
C10—C11	1.369 (2)	C16—H16	0.9298
C11—C12	1.394 (2)	C17—H17	0.9299
C12—C13	1.374 (2)	C18—H18	0.9301
C13—C14	1.384 (2)	C19—H19	0.9297
C15—C16	1.3759 (19)	C20—H20	0.9301
			
C1—O1—C2	117.99 (13)	C18—C19—C20	120.33 (15)
C8—N1—C14	106.60 (10)	C15—C20—C19	119.28 (13)
C8—N1—C15	127.76 (11)	O1—C1—H1A	109.47
C14—N1—C15	125.22 (11)	O1—C1—H1B	109.47
C8—N2—C9	105.19 (11)	O1—C1—H1C	109.47
O1—C2—C3	115.69 (12)	H1A—C1—H1B	109.48
O1—C2—C7	125.03 (13)	H1A—C1—H1C	109.47
C3—C2—C7	119.28 (13)	H1B—C1—H1C	109.47
C2—C3—C4	120.41 (13)	C2—C3—H3	119.80
C3—C4—C5	120.90 (13)	C4—C3—H3	119.80
C4—C5—C6	118.05 (13)	C3—C4—H4	119.56
C4—C5—C8	121.88 (13)	C5—C4—H4	119.55
C6—C5—C8	120.07 (12)	C5—C6—H6	119.33
C5—C6—C7	121.34 (13)	C7—C6—H6	119.33
C2—C7—C6	119.99 (14)	C2—C7—H7	120.01
N1—C8—N2	112.60 (12)	C6—C7—H7	120.00
N1—C8—C5	122.28 (11)	C9—C10—H10	120.82
N2—C8—C5	125.12 (12)	C11—C10—H10	120.81
N2—C9—C10	130.33 (12)	C10—C11—H11	119.36
N2—C9—C14	110.52 (11)	C12—C11—H11	119.33
C10—C9—C14	119.16 (13)	C11—C12—H12	119.14
C9—C10—C11	118.37 (13)	C13—C12—H12	119.12
C10—C11—C12	121.31 (15)	C12—C13—H13	121.75
C11—C12—C13	121.75 (15)	C14—C13—H13	121.74
C12—C13—C14	116.50 (13)	C15—C16—H16	120.49
N1—C14—C9	105.09 (12)	C17—C16—H16	120.46
N1—C14—C13	131.99 (12)	C16—C17—H17	119.69
C9—C14—C13	122.91 (12)	C18—C17—H17	119.68
N1—C15—C16	119.51 (12)	C17—C18—H18	120.02
N1—C15—C20	119.77 (11)	C19—C18—H18	120.00
C16—C15—C20	120.72 (12)	C18—C19—H19	119.83
C15—C16—C17	119.05 (14)	C20—C19—H19	119.83
C16—C17—C18	120.63 (15)	C15—C20—H20	120.35
C17—C18—C19	119.98 (14)	C19—C20—H20	120.37
			
C1—O1—C2—C3	−176.41 (14)	C8—C5—C6—C7	177.52 (13)
C1—O1—C2—C7	2.8 (2)	C4—C5—C8—N1	47.29 (19)
C15—N1—C8—N2	−173.11 (12)	C4—C5—C8—N2	−132.70 (15)
C14—N1—C8—C5	179.73 (12)	C4—C5—C6—C7	−1.9 (2)
C8—N1—C14—C9	0.04 (14)	C6—C5—C8—N2	47.9 (2)
C8—N1—C15—C16	−120.52 (15)	C5—C6—C7—C2	1.0 (2)
C14—N1—C15—C16	67.90 (18)	C14—C9—C10—C11	0.3 (2)
C8—N1—C15—C20	59.48 (18)	C10—C9—C14—C13	−0.6 (2)
C14—N1—C15—C20	−112.11 (15)	N2—C9—C14—N1	0.19 (15)
C15—N1—C8—C5	6.9 (2)	C10—C9—C14—N1	−179.49 (12)
C15—N1—C14—C9	173.11 (12)	N2—C9—C10—C11	−179.29 (14)
C8—N1—C14—C13	−178.74 (15)	N2—C9—C14—C13	179.12 (13)
C15—N1—C14—C13	−5.7 (2)	C9—C10—C11—C12	−0.2 (2)
C14—N1—C8—N2	−0.28 (15)	C10—C11—C12—C13	0.3 (2)
C8—N2—C9—C10	179.28 (14)	C11—C12—C13—C14	−0.5 (2)
C9—N2—C8—C5	−179.62 (13)	C12—C13—C14—N1	179.23 (14)
C9—N2—C8—N1	0.39 (15)	C12—C13—C14—C9	0.6 (2)
C8—N2—C9—C14	−0.35 (15)	N1—C15—C16—C17	179.30 (13)
C7—C2—C3—C4	−2.1 (2)	C16—C15—C20—C19	0.5 (2)
C3—C2—C7—C6	1.0 (2)	C20—C15—C16—C17	−0.7 (2)
O1—C2—C7—C6	−178.14 (13)	N1—C15—C20—C19	−179.51 (13)
O1—C2—C3—C4	177.13 (13)	C15—C16—C17—C18	0.2 (2)
C2—C3—C4—C5	1.2 (2)	C16—C17—C18—C19	0.5 (3)
C3—C4—C5—C8	−178.60 (13)	C17—C18—C19—C20	−0.7 (3)
C3—C4—C5—C6	0.8 (2)	C18—C19—C20—C15	0.2 (2)
C6—C5—C8—N1	−132.09 (14)		

### Hydrogen-bond geometry (Å, º)

Cg2 and Cg3 are the centroids of the C2–C7 and C9–C14 phenyl rings, respectively.

**Table:**

*D*—H···*A*	*D*—H	H···*A*	*D*···*A*	*D*—H···*A*
C6—H6···*Cg*2^i^	0.93	2.86	3.5361 (15)	130
C13—H13···*Cg*3^ii^	0.93	2.83	3.4594 (16)	126

Symmetry codes: (i) −*x*+1, *y*+1/2, −*z*+1/2; (ii) −*x*+2, *y*+1/2, −*z*+1/2.
